# Red Foxes (*Vulpes vulpes*) Are Exposed to High Diversity of *Borrelia burgdorferi* Sensu Lato Species Infecting Fox-Derived *Ixodes* Ticks in West-Central Poland

**DOI:** 10.3390/pathogens11060696

**Published:** 2022-06-16

**Authors:** Beata Wodecka, Jerzy Michalik, Renata Grochowalska

**Affiliations:** 1 Department of Genetics and Genomics, Institute of Biology, University of Szczecin, 71-412 Szczecin, Poland; 2 Department of Animal Morphology, Faculty of Biology, Adam Mickiewicz University, 61-712 Poznań, Poland; jerzy.michalik@amu.edu.pl; 3 Department of Biotechnology, Faculty of Biological Sciences, University of Zielona Góra, 65-516 Zielona Góra, Poland; r.grochowalska@wnb.uz.zgora.pl

**Keywords:** *Ixodes kaiseri*, *Ixodes canisuga*, *Ixodes hexagonus*, red fox, *Borrelia* spp.

## Abstract

The role of red fox, *Vulpes vulpes*, and its associated ticks in maintaining *Borrelia burgdorferi* sensu lato (s.l.) was studied. A total of 1583 ticks were removed from ears of 120 infested animals and were identified as species using a nested PCR targeting the ITS2 and *coxI* fragments of *Ixodes* DNA. *Ixodes kaiseri* prevailed (76%), followed by *I. canisuga*, *I. ricinus*, and *I. hexagonus*. In total, 32.4% of 943 ticks revealed *Borrelia* DNA and 10 species of *B. burgdorferi* s.l. complex were identified. *Borrelia garinii* and *B. afzelii* comprised 70% of all infections. The other eight species included *B. americana, B. bissettiae*, *B. burgdorferi* sensu stricto (s.s.), *B. californiensis, B. carolinensis*, *B. lanei*, *B. spielmanii*, and *B. valaisiana*. Analysis of tissues from 243 foxes showed that 23.5% were infected with *B. burgdorferi* s.l. *Borrelia garinii* was detected in 91% of the infected animals, including 31% of mixed infections with *B. afzelii*, the second most prevalent species, followed by *B. spielmanii*. The predominance of *B. garinii* in PCR-positive animals and infected larval ticks (38.1%), suggests that this spirochete and *B. afzelii* are preferentially associated with foxes. Although red foxes are exposed to a high diversity of *B. burgdorferi* s.l. species found in engorged *Ixodes* ticks, their reservoir competence for most of them appears to be low.

## 1. Introduction

The red fox *Vulpes vulpes* is the most widely distributed terrestrial carnivore species, with its native range including temperate and subarctic regions of the Northern Hemisphere [1]. The implementation of anti-rabies vaccination during the past three decades increased population density of foxes in Central Europe, including Poland [2]. In contrast to cervids or wild boars, the red fox is a game animal hunted predominantly as a pest, mostly in farmland areas without the emphasis on trophy hunting [1]. In Poland, according to the Polish Hunters Association, the red fox is the most abundant carnivore, with an annual harvest estimated at approximately 150,000 animals.

Foxes are hosts for hematophagous ectoparasites, primarily fleas and ixodid ticks, transmitting a wide variety of blood-borne bacterial agents [3,4,5,6]. European eco-epidemiological studies on the identification of potentially zoonotic vector-borne bacteria in foxes, documented the presence of *Anaplasma phagocytophilum* [7,8], *A. platys*, *Ehrlichia canis* [9,10], and *Candidatus* Neoehrlichia sp. (FU98) [11]. Moreover, *Bartonella rochalimae* was confirmed in red foxes sampled in Spain [12,13]. However, the role of the red fox in the epidemiology of these vector-borne agents is still poorly understood. This concerns the spirochete species from the complex of *Borrelia burgdorferi* sensu lato (s.l.), some of which are the causative agents of Lyme disease (LD). To date, there are only a few European reports on the occurrence of *B. burgdorferi* s.l. in tissues of red foxes sampled in Germany [14,15], Romania [16], and Norway [17]. These carnivores may host all of the developmental stages of *Ixodes ricinus*, the primary vector of LD spirochetes and, additionally, all instars of three nidicolous *Ixodes* species: *I. canisuga*, *I. hexagonus*, and *I. kaiseri* [5,18]. The co-occurrence of these three host-specific tick species on the red fox could potentially increase diversity of the *Borrelia* species, which may be transferred to local populations of the generalist tick *I. ricinus* acting as a bridge vector to humans. However, so far, the involvement of the burrow-dwelling *Ixodes* species in the ecology of *Borrelia* species and other tick-borne pathogens is limited mainly to *I. hexagonus* that has been demonstrated as a competent vector for LD spirochetes [19]. Moreover, *B. burgdorferi* s.l. infection was also found in *I. canisuga* ticks removed from foxes in north-eastern Spain [20]. Recently, based on molecular tick species identification, we have confirmed the presence of the bacterium in the three carnivore-associated ticks: *I. canisuga, I. hexagonus,* and *I. kaiseri* collected from the racoon dog *Nyctereutes procyonoides* and the European badger *Meles meles* [21]. These sparse reports seem to result from difficulties in the accurate morphological identification of tick species, especially their immature stages, which prevail on carnivorous mammals. In the present study, we report the results of a three-year study on the role of red foxes and their ticks in the ecology of *Borrelia* spirochetes.

## 2. Material and Methods

### 2.1. Study Area and Sample Collection

**Figure 1:**
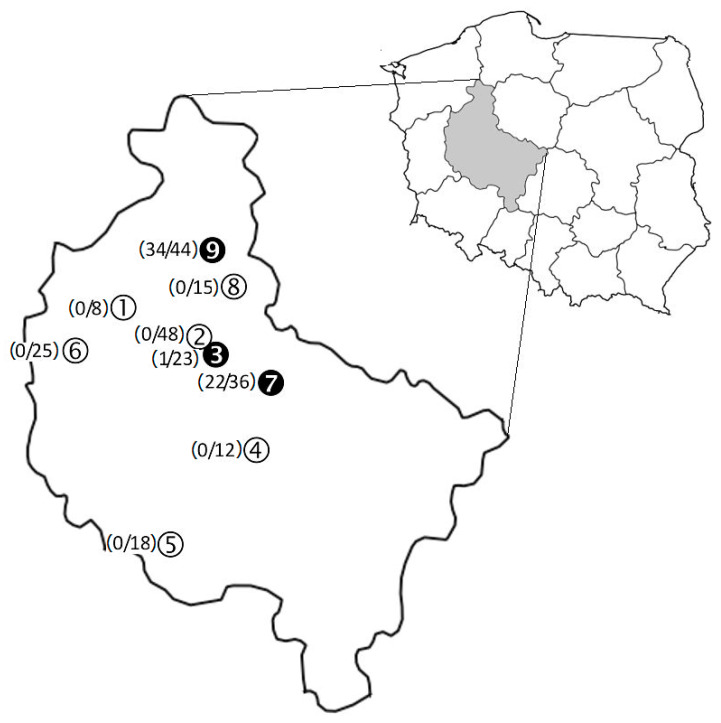
Location of nine collection sites where red foxes were harvested in Wielkopolska province, west-central Poland: six Forest Ranges: (1) Annogóra, Lubasz Commune: 52°51′ N 16°31′ E; (2) Kąty, Murowana Goślina Commune: 52°37′22″ N 17°00′22″ E; (3) Zielonka, Murowana Goślina Commune: 52°33′00.0″ N 17°07′00.0″ E; (4) Murzynówko, Krzykosy Commune: 52°09′26″ N 17°23′18″ E; (5) Poniec Commune: 51°45′50″ N 16°48′30″ E; (6) Niemierzewo, Kwilcz Commune: 52°33′50″ N 16°10′12″ E), and three Forest Districts: (7) Czerniejewo Commune: 52°26′ N 17°29′ E; (8) Durowo, Wągrowiec Commune 52°48′25″ N 17°12′25″ E; (9) Margonin Commune: 52°58′23′89″ N 17°05′41′27″ E. In brackets: number of PCR positive hosts vs. tested. Black circles denote collection sites with infected foxes. Animals were harvested during fox-hunting seasons (1 June to 31 March) from 2009 to 2011. *Borrelia garinii* was found in fox-derived ticks in sites no. 1–7 and 9, *B. afzelii* in sites 1–4 and 6–9, *B. burgdorferi* s.s. in sites no. 1, 3, 4, 6, and 7, *B. valaisiana* in sites no. 6, 7, and 9, *B. spielmanii* in sites no. 1, 6, 7, and 9, *B. bissettiae* in sites no. 1, 6, and 7; *B. carolinensis* and *B. californiensis* in sites no. 6, 7, and 9; *B. lanei* in sites no. 6, and 9; *B. americana* in site no. 7, and *B. turcica* in site no. 6.

Foxes were derived from six forest ranges and three forest districts, all situated in Wielkopolska province in west-central Poland. Their localization is shown in Figure 1.

Most of the foxes were harvested in agricultural landscapes neighbouring various forest ecosystems during the fox-hunting seasons (1 June to 31 March) from 2009 to 2011. Autumn and winter are preferred seasons for fox hunting in Poland. Infestation parameters, such as mean prevalence, abundance, and intensity, were estimated for each *Ixodes* species. Seasons were grouped as (i) spring–summer: June–August, (ii) autumn: September–November, and (iii) winter: December–March. Comparable numbers of foxes were obtained from each of the three consecutive seasons (70, 82, and 77, respectively).

In Poland most of the hunted foxes are left in the hunting ground and buried so acquired material was limited only to blood and biopsies of skin and liver collected by the hunters. In total, 558 tissue samples were obtained from 243 foxes. EDTA-whole blood was collected from the body cavity out of 216 (88.9%) of the animals tested. Furthermore, biopsies of skin (from ear) and liver were obtained from 243 and 99 of the foxes, respectively. These three types of tissue samples were collected within one hour after each animal was culled and stored at −20 °C until extraction.

Both ears of 243 red foxes were cut off basally by hunters and placed into a separate plastic bag, and stored at −20 °C. In the laboratory, after thawing, ears were inspected for ticks under a stereoscopic microscope. Ticks were counted and stored in plastic vials containing 75% ethanol. A total of 1583 *Ixodes* ticks were removed from 120 infested animals. Since morphological identification of adult female ticks to the species level is easier than immature ticks, females were determined by using taxonomic keys by Siuda [22] and Hornok et al. [18]. Molecular procedures were used for the species determination of larval and nymphal stages.

### 2.2. DNA Extraction

DNA extraction from animal tissues and engorged ticks was performed with a phenol–chloroform protocol [23]. To avoid any contamination only undamaged ticks rinsed with 75% ethanol before DNA extraction were selected for bacterial DNA detection. DNA samples were stored at −70 °C before PCR analyses.

### 2.3. Molecular Identification of Ixodes Tick Species

To confirm the accuracy of morphological tick identification according to taxonomic keys, nested PCR assays based on two molecular markers and primer sets targeting (i) the ITS2 (internal transcribed spacer 2) fragment from the nuclear genome of Ixodidae [21], and (ii) the mitochondrial cytochrome c oxidase subunit I (*coxI*) were used (Table 1).

In each PCR run, DNA isolates of four reference *Ixodes* spp. female ticks—*I. hexagonus* from the European hedgehog *Erinaceus europaeus*, *I. canisuga* and *I. kaiseri* from the racoon dog, and *I. ricinus* from the vegetation—were used. The identities of these ticks had been previously validated with morphological taxonomic keys and molecular studies [18,21,22]. The PCR products were separated on 1.5% agarose gel (Bioshop, Burlington, ON, Canada) and archived, as described elsewhere [21].

In the first stage, including all tick specimens, molecular identification was performed, based on PCR-restriction fragment length polymorphism analysis (PCR-RFLP). PCR-amplified sequences of the ITS2 gene generated with primers SP2-100f and SP2-1274r were digested with enzyme AluI (Thermo Fisher Scientific, Waltham, MA, USA) to obtain RFLP patterns for different *Ixodes* species according to the protocol previously described [21]. In the second stage, to validate identification done by PCR-RFLP analysis of the ITS2 gene, partial sequencing of *Ixodes* DNA of ITS2 fragments amplified with inner primer sets SP-100f/SP-1274r was performed for subset of amplicons representing different restriction patterns. Furthermore, partial sequencing of the *coxI* gene fragments of *Ixodes* obtained with inner primer sets CO1-375f/CO1-1086r was performed (Table 1). Sequencing was conducted in Macrogen Europe (The Netherlands). Representative partial sequences (*n* = 78) were deposited in GenBank. The *ITS2* sequences (*n* = 43) are listed as follows: MG962859–MG962867 (*I. kaiseri*), MG962868–MG962875 (*I. hexagonus*), MG962876–MG962886 (*I. canisuga*), and MG962887–MG962901 (*I. ricinus*). The *coxI* sequences (*n* = 35) are listed as follows: MH109183–MH109187 (*I. canisuga*), MH109188–MH109204 (*I. ricinus*), MH109205–MH109212 (*I. hexagonus*), and MH109213-MH109217 (*I. kaiseri*).

### 2.4. Detection of Borrelia DNA by Nested PCR

Altogether, 943 undamaged, fully, or partially, engorged ticks (76 females, 166 nymphs, and 701 larvae) and the 558 tissues from foxes were selected. Both groups of samples were tested for the presence of *Borrelia* DNA using a nested PCR with two primer sets amplifying a fragment of the *flaB* gene; the protocol has been previously described by Wodecka et al. [24]. All positive samples were rerun using nested PCR assays with two independent sets of primers targeting parts of the *p66* gene and intergenic spacer between 3-methyladenine glycosylase (*mag*) gene and *trnI* gene encoding tRNA for isoleucine (Table 1). To confirm the specificity and sensitivity of implemented protocols in each PCR run, randomly selected DNA isolated from one of the 11 reference strains of *Borrelia* spp. was used as a positive control and TE buffer was used as a negative control. In total, 11 reference strains were included: *B. burgdorferi* sensu stricto (s.s.) IRS, *B. garinii* 20047, *B. afzelii* VS461, *B. valaisiana* VS116, *B. bissettiae* DN127, *B. spielmanii* PC-Eq17, *B. californiensis* CA446, *B. carolinensis* SCW-22, *B. lanei* CA28, *B. americana* CA8, and *B. turcica* IST7 (German Collection of Microorganisms and Cell Cultures—DSMZ, Germany). The PCR products were separated on 1.5% agarose gel and the results were written, as described elsewhere [21].

### 2.5. Identification of Borrelia Species by PCR-RFLP and Sequencing

The *flaB* gene fragments amplified with primers 220f and 823 r were digested with enzymes HpyF3I and Ecl136II (Thermo Fisher Scientific, Waltham, MA, USA) to obtain RFLP patterns of different *Borrelia* species, according to the protocol previously described [25]. To confirm *Borrelia* species identification based on PCR-RFLP analysis, partial sequencing of the *flaB* gene products amplified with primers 220f and 823r or FL120F and FL908R, *p66* gene fragments obtained with primers P66-487F and P66-1087R and fragments of intergenic spacer between *mag* gene and *trnI* gene generated with primers glz435F and ile65R (Table 1) was performed for positive amplicons, representing different restriction patterns. DNA sequencing was performed in Macrogen Europe (Amsterdam, The Netherlands). Obtained sequences were compared with those available in the GenBank databases using BLAST program (US National Institutes of Health, Bethesda, MD, USA) [www.ncbi.nlm.nih.gov/blast/Blast.cgi (accessed on 23 March 2018)]. A total of 189 selected sequences of the *flab* gene (*n* = 112), the *p66* gene (*n* = 33), and the *mag*—*trnI* intergenic spacers (*n* = 44) of *Borrelia* spp. were deposited in GenBank.

The *flaB* gene sequences are listed as follows: HM802182, HM802184–HM802185, HM802188, KF422758–KF422759, KF422762–KF422767, KF422774, KF422779–KF422782, KF422817, KF422838–KF422846, KF918600–KF918612, MG944996 (*B. garinii*), HM802193, KF422787, KF422789–KF422791, KF422794–KF422796, KF422858–KF422865, KF918614–KF918616, MG944961–MG944963 (*B. afzelii*), HM802191, KF422799, KF422802, KF918617, MG944978–MG944983 (*B. burgdorferi* s.s.), MT118979–MT118980 (*B. valaisiana*), KF422806–KF422807, KF918618, MG944964 (*B. bissettiae*), JF732881, MG944976–MG944977, MT118981–MT118982 (*B. spielmanii*), MG944984–MG944995 (*B. californiensis*), MG944970–MG944975 (*B. carolinensis*), MG944965–MG944969 (*B. lanei*), KF918619–KF918622 (*B. americana*), and MG944997 (*B. turcica*).

The *p66* gene sequences are listed as follows: MT118983–MT118984 (*B. garinii*), MT118985–MT118987 (*B. afzelii*), MT118988–MT118991 (*B. burgdorferi* s.s.), MT118992–MT118993 (*B. valaisiana*), MT118998 (*B. bissettiae*), MT118994–MT118997 (*B. spielmanii*), MT119004–MT119007 (*B. californiensis*), MT118999–MT119002 (*B. carolinensis*), MT119009–MT119013 (*B. lanei*), and MT119015–MT119018 (*B. americana*).

The *mag*–*trnI* intergenic spacer sequences are listed as follows: MT119020–MT119023 (*B. garinii*), MT119024–MT119029 (*B. afzelii*), MT119030–MT119034 (*B. burgdorferi* s.s.), MT119035–MT119036 (*B. valaisiana*), MT110941–MT119042 (*B. bissettiae*), MT119037–MT119040 (*B. spielmanii*), MT119050–MT119054 (*B. californiensis*), MT119043–MT119048 (*B. carolinensis*), MT119056–MT119060 (*B. lanei*), MT119062–MT119065 (*B. americana*), and MT119067 (*B. turcica*).

### 2.6. Contamination Control in DNA Analysis

To minimize contamination, the processes of DNA isolation, the reaction mixture preparation and electrophoresis were carried out in separate rooms.

### 2.7. DNA Sequence Analysis

Aligned sequences representing 43 nuclear *ITS2* gene fragments and 35 mitochondrial *coxI* gene fragments of *Ixodes* strains as well as 112 *flaB* gene, 33 *p66* gene, and 44 *mag–trnI* intergenic spacer fragments of *Borrelia* strains were examined with MEGAX software (Molecular Evolutionary Genetics Analysis, version X) [26]. Relationships between individuals were assessed by distance estimation between sequences as a measure of the number of allelic substitutions on selected loci, as described earlier [21].

### 2.8. Statistical Analyses

We analysed *Borrelia* prevalence in ticks using chi-squared test with Yates’ correction. Differences in mean intensity of tick infestation by Mann–Whitney U test with *p* < 0.05 were considered statistically significant. All calculations were done using Statistica 8.0 software (StatSoft Inc., Palo Alto, CA, USA).

## 3. Results

### 3.1. Tick Species Identification

Of the 243 red foxes, 120 (49.4%) hosted on their ears 1583 *Ixodes* ticks (Table 2). Using morphological criteria for tick females, four *Ixodes* species were identified: *I. ricinus*, *I. kaiseri, I. canisuga*, and *I. hexagonus*. Analysis of ITS2 DNA fragments of these females according to the method described by Wodecka et al. [21], confirmed morphological identification.

Analysis of 43 ITS2 sequences obtained from ticks representing different PCR products or restriction patterns, revealed also four distinct groups represented by *I. kaiseri* (*n* = 9), *I. canisuga* (*n* = 11), *I. hexagonus* (*n* = 8), and *I. ricinus* (*n* = 15). Comparative analysis of these sequences generated mean distance values within each species as follows: 0.0 for *I. canisuga*, *I. kaiseri*, and *I. hexagonus*, and 0.023 for *I. ricinus*. The analysis of mean distance values between species showed the highest value for *I. ricinus* and the three nidicolous tick species: *I. canisuga*, *I. kaiseri*, and *I. hexagonus* (0.262, 0.387, and 0.332, respectively). The distance among these ticks reached: 0.118 for *I. kaiseri* and *I. hexagonus*, 0.126 for *I. canisuga* and *I. hexagonus,* and 0.185 for *I. canisuga* and *I. kaiseri*.

To confirm the ITS2 identification of tick species and its conformity with morphological characterization according to a key by Hornok et al. [18], the analysis of 35 *coxI* sequences was carried out. This analysis confirmed four distinct groups represented by *I. kaiseri* (*n* = 5), *I. canisuga* (*n* = 5), *I. hexagonus* (*n* = 8), and *I. ricinus* (*n* = 17). Comparative analysis of these sequences revealed different mean distance values within each species as represented ITS2 sequences analysis: 0.004 for *I. canisuga*, 0.002 for *I. kaiseri*, 0.001 for *I. hexagonus*, and 0.005 for *I. ricinus*. The analysis of mean distance values between species confirmed their molecular distinctiveness and the highest value for the exophilic *I. ricinus* and two nidicolous tick species *I. kaiseri* and *I. hexagonus* (0.306 and 0.316, respectively). Surprisingly the distance value for *I. ricinus* and *I. canisuga* (0.178) was lower than those for *I. canisuga* and *I. kaiseri* (0.212) and *I. canisuga* and *I. hexagonus* (0.226). The distance for *I. kaiseri* and *I. hexagonus* was the lowest (0.142).

### 3.2. Occurrence of Tick Species on Ears of Red Foxes

Out of 1583 *Ixodes* specimens collected from 120 foxes, 1341 were larvae, 166 nymphs, and 76 females. On an average, one infested animal hosted 13.2 ticks (Table 2). Larvae distinctly prevailed over nymphs and females (84.7% vs. 10.5% and 4.8% of total tick numbers, respectively). Larvae infested 34% on animals, nymphs 30%, and females 17%. In total, 55 (45.8%) of the infested animals were parasitized concurrently by at least two of the three different tick developmental stages.

The most abundant tick species was *I. kaiseri* (76.1%), followed by *I. canisuga* (11.9%), *I. ricinus* (10.2%), and *I. hexagonus* (1.8%, Table 2). *Ixodes kaiseri* occurred on 33.7% of foxes with a mean intensity of 14.7 ticks (standard deviation, SD—41.01). *I. canisuga* and *I. ricinus* reached comparable values of mean intensities (4.2/SD 6.21 and 2.9 ticks/SD 2.83, respectively) and infested 18.5% and 23% of animals, respectively. Only 28 of *I. hexagonus* ticks were removed from 7.8% of hosts with a mean intensity of 1.5 ticks (SD 0.75). Co-infestations with two, three, or four different tick species (41 double, 16 triple, and 3 quadruple) were noted for 50% of the infested animals. Over half of the double co-infestations (*n* = 21) was constituted by *I. kaiseri* and *I. ricinus*, whereas *I. kaiseri, I. canisuga,* and *I. ricinus* accounted for half of the triple infestations (*n* = 8). Double and triple co-infestations were more prevalent in autumn (39% and 44%, respectively).

Most *I. kaiseri* (92%, *n* = 1109) and *I. canisuga* (81%, *n* = 152) ticks were larvae which contributed to the larval predominance on the infested foxes. Except for the rare *I. hexagonus*, loads of nymphs were comparatively low for each of the other three tick species as sparser than larvae intermediate stage (range: 0.1–0.3 ticks per animal). Female ticks were represented mostly by *I. ricinus* and *I. kaiseri,* which constituted 84% of the 76 females.

Each of the four *Ixodes* species occurred on foxes year-round (Table S1). *Ixodes ricinus* demonstrated clear seasonality, with intensity and prevalence values decreasing steadily from spring–summer to winter. *Ixodes canisuga* tended to be more prevalent and abundant in winter, whereas the remaining ticks were collected mostly in autumn and winter months. *Ixodes kaiseri* was more abundant in spring–summer and winter.

### 3.3. Detection of Borrelia DNA by Nested PCR in Foxes and Their Ticks

Of the 558 tissue samples obtained from the 243 red foxes, 67 (12%) displayed *Borrelia* spp. specific DNA (Table 3). The bacterium was identified in all three isolate types, but it occurred more frequently in blood samples (23.6%) than in liver (6.1%) or skin (4.1%) biopsies. The infected isolates were derived from 57 (23.5%) of the animals tested. A total of 47 (82.5%) of the infected foxes revealed the bacterium in one isolate type. In this group, blood PCR positive animals (*n* = 42) prevailed over those carrying the pathogen in liver (*n* = 3) or skin (*n* = 2) biopsies. A total of 10 animals (17.5%) harboured spirochetes concurrently in two isolate types: in blood and skin samples (*n* = 6), in blood and liver samples (*n* = 3), and in skin and liver biopsies (*n* = 1, Table S2). The infected foxes were detected in three of the nine sampling sites (Figure 1) with the highest prevalence in Margonin (34/44, 77%) and Czerniejewo (22/36, 61%), and the lowest in Zielonka (1/23, 4.3%).

*Borrelia* DNA was identified in 306 (32.4%) of the 943 ticks tested and occurred in all parasitic life stages (Table 4). Of the 120 infested animals, 81 (67.5%) hosted at least one infected tick. Of the 81 hosts, 46 (56.8%) carried infected larvae. A total of 12 (14.8%) of the foxes carrying infected ticks proved to harbour concurrently *Borrelia* spirochetes. Female and nymphal ticks were infected with the bacterium more frequently than larvae (47.4% and 43.9% vs. 28.1%, respectively; *p* = 0.014). The overall prevalence of infection in *I. ricinus* ticks was comparable to those recorded in three nidicolous tick species.

### 3.4. Identification of Borrelia Species

Altogether, PCR positive for *flaB* gene tissue samples from red foxes and ticks revealed 11 unique RFLP patterns corresponding with different *Borrelia* species. Of the 11 species, 10 were attributed to the *B. burgdorferi* s.l. complex i.e., *B. garinii*, *B. afzelii*, *B. spielmanii*, *B. burgdorferi* s.s., *B. valaisiana*, *B. bissettiae*, *B. carolinensis*, *B. californiensis*, *B. lanei*, and *B. americana*. The first three species were found in the infected foxes, whereas PCR positive ticks revealed all 10 spirochete species (Table 3 and Table 5). Furthermore *B. turcica* of the reptile-associated borreliae (REP) group was found in a single larva of *I. kaiseri*.

*Borrelia garinii*, the most prevalent species infecting foxes, was identified in 52 (21.4%) of the 243 animals tested (36 single and 16 double infections with *B. afzelii*), followed by *B. afzelii* (20/8.2%, 4 single and 16 double infections). Moreover *B. spielmanii* was amplified from one skin sample (Table 3). A total of 10 infected hosts revealed spirochetes concurrently in two different tissue isolate types (Table S2).

Among the 306 *Borrelia* infected ticks, *B. garinii* (112/36.6%, including 12 double infections) and *B. afzelii* (102/33.3%, including 10 double infections) were the most prevalent species (Table 5). The third most frequent species was *B. carolinensis* (36/11.8%), followed by *B. californiensis* (24/7.8%), *B. burgdorferi* s.s. (15/4.9%, including 3 co-infections), *B. spielmanii* (9/2.9%), *B. bissettiae* (8/2.6%), *B. lanei* (5/1.6%), *B. americana* (4/1.3%, including 1 co-infection), *B. valaisiana* (3/1%), and *B. turcica* (1/0.3%). Double infections occurred in 13 (4.2%) of the infected ticks, of which 9 were larvae. Out of the 81 animals carrying at least 1 infected tick, 7 revealed the same spirochete species as their feeding ticks (*B. garinii* and/or *B. afzelii*; Table 6).

All 11 identified *Borrelia* species were detected in *I. kaiseri*, 10 in *I. canisuga* (except for *B. turcica*), 8 in *I. ricinus* (except for *B. bissettiae*, *B. lanei*, and *B. turcica*), and 4 in *I. hexagonus* (*B. garinii*, *B. afzelii*, *B. burgdorferi* s.s., and *B. bissettiae*) (Table 5). The two most prevalent species, namely *B. garinii* and *B. afzelii*, were identified in eight out of the nine collection sites. Furthermore, *B. burgdorferi* s.s. positive ticks were obtained from foxes harvested in five study sites; *B. valaisiana*, *B. spielmanii*, *B.* *bissettiae*, *B. carolinensis*, and *B. californiensis* in three; *B. lanei* in two, whereas *B. americana* and *B. turcica* were detected only in one site (Figure 1).

Subsequent BLAST analysis of 112 selected sequences of the *flaB* gene fragments from tissue samples and ticks confirmed *Borrelia* species identification based on PCR-RFLP and revealed also 11 distinct groups represented by all identified species (Table S3). Comparative analysis of these sequences and reference sequences generated mean distance values within each species as follows: 0.0 for *B. valaisiana*, *B. carolinensis,* and *B. lanei*, 0.001 for *B. spielmanii*, 0.002 for *B. californiensis* and *B. turcica*, 0.003 for *B. bissettiae*, 0.004 for *B. burgdorferi* s.s., 0.005 for *B. americana*, 0.013 for *B. afzelii,* and 0.018 for *B. garinii*. The analysis of mean distance values between species showed the highest value for *B. turcica* and 10 Lyme disease species (values from 0.21 to 0.245, Table S4). The distance among LD borreliae species reached values from 0.006 for *B. bissettiae* and *B. carolinensis* to 0.075 for *B. garinii* and *B. burgdorferi* s.s. (Table S4).

The analysis of 33 sequences of the *p66* gene obtained with *B. burgdorferi* s.l. specific primers confirmed 10 distinct groups of this complex (Table S5). Comparative analysis of obtained and reference sequences revealed different mean distance values within each species, as represented in the case of *flaB* gene: 0.004 for *B. spielmanii*, 0.005 for *B. lanei*, 0.006 for *B. valaisiana*, 0.01 for *B. californiensis*, 0.011 for *B. burgdorferi* s.s., *B. afzelii* and *B. americana*, 0.012 for *B. carolinensis*, and 0.015 for *B. garinii* and *B. bissettiae*. The analysis of mean distance values between LD borreliae species confirmed their molecular distinctiveness and revealed the distance values ranged from 0.012 for *B. afzelii* and *B. spielmanii* to 0.121 for *B. valaisiana* and *B. carolinensis* (Table S6).

The analysis of the 44 sequences of *mag*–*trnI* intergenic spacer confirmed all 11 identified spirochete species (Table S7). Comparative analysis of these sequences revealed not only different mean distance values within each species, as represented by the *flaB* and *p66* gene sequences analysis, but also differences in length of sequence, depending on *Borrelia* species (Table S7). Mean distance values within each species are as follows: 0.0 for *B. valaisiana*, 0.002 for *B. spielmanii*, 0.003 for *B. burgdorferi* s.s., *B. californiensis,* and *B. turcica*, 0.004 for *B. afzelii* and *B. lanei*, 0.007 for *B. bissettiae*, 0.008 for *B. americana*, 0.012 for *B. carolinensis*, and 0.014 for *B. garinii*. The analysis of mean distance values between species demonstrated the highest values of all examined molecular markers. The highest values were obtained for *B. turcica* and 10 Lyme disease borreliae species (values from 0.539 to 0.631, Table S8). The distance among LD species reached values from 0.074 for *B. lanei* and *B. americana* to 0.31 for *B. spielmanii* and *B. bissettiae* (Table S8).

## 4. Discussion

Based on morphological and molecular methods, we documented the occurrence of four *Ixodes* tick species on the ears of red foxes. Furthermore, we provide evidence of *Borrelia* spp. infections in tissue samples of red foxes and in their *Ixodes* ticks collected from the ears.

### 4.1. Ixodes Tick Species on Red Foxes

We confirmed the presence of three nidicolous *Ixodes* species of the subgenus *Pholeoixodes*, namely *I*. *kaiseri*, *I*. *canisuga*, *I*. *hexagonus*, and the generalist exophilic *I. ricinus* on red foxes. The first three species are considered nonhuman-biting and are associated mainly with Canidae and Mustelidae and reproduce inside the burrows of their hosts [22,27]. *Ixodes hexagonus* infests hedgehogs also those inhabiting sub- and urban areas [28]. A taxonomic key published by Hornok et al. [18] allowed us to identify *Pholeoixodes* females of the three tick species collected in our study. Furthermore, comparison of the *coxI* sequences obtained from the nidicolous tick species with those published by Hornok et al. [18] confirmed high reliability of this molecular marker in accuracy of species identification of ticks. Therefore, using the *coxI* gene, we re-examined feeding ticks derived from raccoon dogs and badgers tested in our previous study [21]. This new analysis showed that engorged females of *I. kaiseri* have been misnamed on the basis of morphological analysis and ITS2 reference sequences deposited in GenBank database as *I. canisuga* and vice versa. As it turned out, *I. kaiseri,* and not *I. canisuga,* dominated both on raccoon dogs and badgers (44.8% vs. 14% and 48.2% vs. 18.5%, respectively). Our present study confirmed the importance of *I. kaiseri* as the most prevalent tick species associated with wild carnivores in west-central Poland. Moreover, we suggest that in Central Europe, *I. kaiseri* parasitizes these mammals more frequently than previously thought and its former distribution is not restricted to Romania, the Republic of Moldavia, and Ukraine [22,29]. This confirms the report by Hornok et al. [18] in which the presence of *I. kaiseri* was documented on carnivores from Germany, Hungary, Romania, and Serbia, expanding its known geographical range in Europe. In our opinion, its hitherto infrequent occurrence on European carnivore species results from misidentification of the three nidicolous *Ixodes* species using only the morphological approach, therefore, molecular verification is necessary to avoid any misidentification between morphologically similar *Ixodes* species of the subgenus *Pholeoixodes*.

### 4.2. Borrelia Infections in Red Foxes

We found that overall, 23.5% of red foxes revealed DNA of *Borrelia* spp. Interestingly, over 92% of the 51 blood PCR positive hosts (including 21.3% double infections with *B. afzelii*) carried *B. garinii*. Infections caused by *B. garinii* were also found in skin and liver biopsies. This finding indicates that red foxes develop a disseminated infection with *B. garinii* more frequently than with *B. afzelii,* known to exhibit the host specificity for mammals [30]. We also found a predominance of *B. garinii* over *B. afzelii* (62.5% vs. 25%) in PCR-positive raccoon dogs [21]. Co-occurrence of both spirochete species has been reported in one European hedgehog *Erinaceus europaeus* [31] and one Siberian chipmunk *Tamias sibiricus barberi* [32]. Dumitrache et al. [16] found only *B. afzelii* (*n* = 4) and *B. burgdorferi* s.s. (*n* = 1) in 1.4% of 353 fox heart isolates tested in Romania. In a German study 24% of skin samples of red foxes had only *B. garinii* [14]. Furthermore, evidence of *B. garinii* in blood of a dog from the Czech Republic [33] and in two dogs in Japan was reported [34]. Therefore, we suggest that this avian-adapted spirochete seems to be often associated with canids, including the red fox. However, to clarify its status as a potential reservoir for *B. garinii*, xenodiagnostic experiments are necessary.

### 4.3. Borrelia Infections in Ixodes Ticks

The overall infection prevalence found in the four *Ixodes* tick species collected from ears of red foxes was 32.4% and ranged from 30% to 37%, depending on tick species. The PCR-positive ticks were found year-round. A total of 10 species of the *B. burgdorferi* s.l. complex were identified, and *I. ricinus* was infected with eight species, whereas their number in the burrow-dwelling ticks ranged from four (*I. hexagonus*) to 10 (*I. canisuga,* and *I. kaiseri*). So far, there is only one report from Switzerland in *I. ricinus* ticks infected with eight *B. burgdorferi* s.l. species [35]. To our knowledge, none of the European mammals has been found to be exposed to such diversity of *B. burgdorferi* s.l. species as we report here for fox-derived ticks.

**Figure 2:**
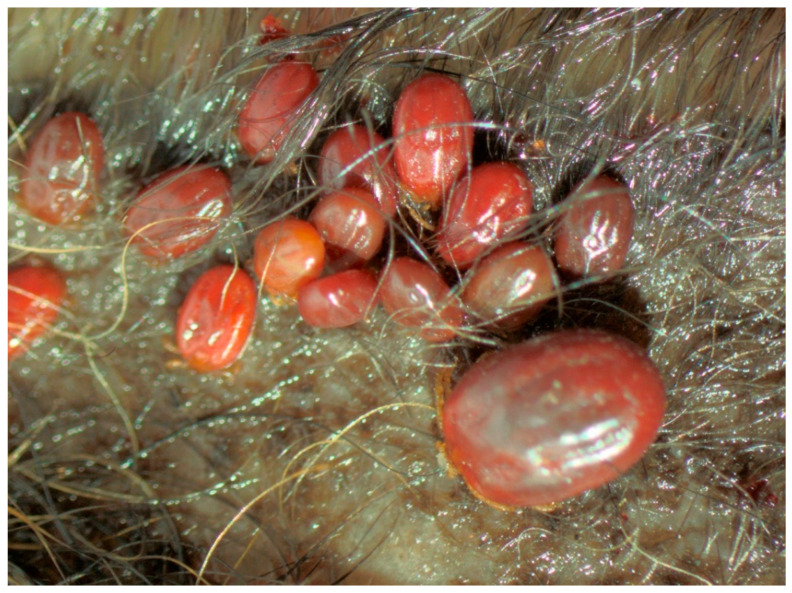
Larvae co-feeding with a nymph of *Ixodes kaiseri* on an ear tissue of the red fox (Michalik J.).

Similarly, as in the case of the PCR-positive foxes, *B. garinii* and *B. afzelii* comprised most (70%) of the total infections, and reached comparable infection prevalence in each of the four tick species. The distinct predominance of *B. garinii* and *B. afzelii* in infected larvae, as well as in foxes, suggests that larvae could have acquired infection while feeding on animals infected with these spirochete species, especially that larval ticks are rarely infected transovarially with *B. burgdorferi* s.l. Interestingly, the most abundant *B. afzelii* and *B. garinii* are associated with rodents and birds, respectively [30], the vertebrates serving as the main food source for red foxes [1]. Therefore, their abundance in our study is not a surprise, especially in the case of *I. ricinus* that may be shared by the fox and its prey. The presence of the remaining eight species in fox-derived ticks and their absence in hosts, might be explained by co-feeding transmission between spatially clustered infected and uninfected ticks [36]. The observed high aggregation of larval ticks on the ears of the foxes, and the fact that nearly half (46%) of the infested animals were parasitized concurrently by larvae and nymphs or females could greatly enhance the efficiency of this localized non-systemic transmission (Figure 2). Furthermore, the finding that in the case of 11 non-infected foxes, larvae co-feeding with nymphs or females carried the same spirochete species supports our assumption (Table 6). This mode of transmission may facilitate contact and exchange between *Borrelia* species adapted to different vertebrate host species [37].

Surprisingly, four *B. burgdorferi* s.l. species previously believed to be restricted only to North America: *B. carolinensis, B. californiensis, B. americana*, and *B. lanei* were identified. The first two species were the most prevalent in PCR-positive ticks after *B. garinii* and *B. afzelii* and constituted almost 20% of all infections. To date, there are only two European reports documenting the presence of *B. carolinensis* in ticks: first in one (2.9%) questing *I. ricinus* from France [38], and the second case together with *B. lanei* detected for the first time in Europe in three *Ixodes* tick species associated with European bats from Poland and Romania [39]. In South Carolina and California enzootic cycles of *B. carolinensis* involve small rodents and a nidicolous *I. minor* [40,41]. *Borrelia californiensis* is maintained by Cali–fornia kangaroo rats *Dipodomys californicus* and two non-human (or rarely) biting *I. jellisoni* and *I. spinipalipis* as well as *I. pacificus,* the key vector of LD species in the western United States [42]. Importantly, in our study *B. carolinensis* and, for the first time in Europe, *B. californiensis* were also detected in PCR-positive *I. ricinus* ticks. Unexpectedly, we detected sequences almost identical to California strains of *B*. *americana* in two females of *I. ricinus* and individual nymphs of *I. cansiuga* and *I. kaiseri*. This spirochete was first identified in *I. minor* from birds in South Carolina as well as from *I. pacificus* in California [43]. Outside the United States, it was detected only in *I. persulcatus* ticks in China [44] and, recently, in two *I. ricinus* specimens in Poland [45]. Thus, our study reaffirms that *B. americana* occurs in *Ixodes* ticks in three continents, but its vertebrate host(s) in Europe remains unknown. We identified *B. lanei* in *I. kaiseri* and *I. canisuga*. Considering the study by Michalik et al. [39], it is the second report documenting *B. lanei* in the group of European *Ixodes* ticks inhabiting the burrows or caves of their hosts. In the USA this spirochete perpetuates in enzootic cycles involving lagomorphs and rodents and the two tick species, of which *I*. *spinipalpis* is nidicolous [46,47].

*Borrelia bissettiae* like *B. burgdorferi* s.s., occurs in both the Old and the New World and is considered a human pathogen in Europe and a likely one in the United States [48,49]. In the United States transmission cycles of this species are driven by *I. pacificus,* and three nidicolous species, *I. spinipalpis, I. minor,* and *I. affinis,* and various rodent species acting as reservoirs [42,50,51], and has been detected in the blood of several bird species and *I. pacificus* feeding on them [52]. Our findings suggest that three burrow-dwelling tick species associated with the red fox could similarly serve as enzootic maintenance vectors of this rarely recorded spirochete in Europe, for which prevalence in *I. ricinus* is very low [53,54,55]. Furthermore, the finding that *B. bissettiae* infected ticks were collected only in three collection sites, confirms its highly focal distribution. Although mixed infections of *B. bissettiae* and *B. carolinensis* were found in tissues from the European hedgehog, and the Eurasian red squirrel *Sciurus vulgaris* from the Czech Republic [56], any association of this spirochete with particular vertebrate host(s) in Europe is speculative, at best.

*Borrelia spielmanii,* with pathogenic potential to humans [57], was detected with a low 1% total prevalence, including six larvae of *I. kaiseri,* two *I. canisuga*, and a single *I. ricinus* female. Together with *B. afzelii* and *B. bavariensis* it is considered as maintained by small- and medium-sized mammals [58,59]. Interestingly, it prevailed in bat specific *Ixodes* ticks [39] and was also reported in bird-derived ticks [60,61].

Enigmatic is evidence of *B. turcica* in a single larva of *I. kaiseri*. This spirochete was originally isolated from *Hyalomma aegyptium* ticks associated with Mediterranean *Testudo* tortoises in Turkey [62] and is a member of the reptile-associated borreliae (REP), representing the third major group of spirochetes, distinct from LD and relapsing fever (RF) borreliae [63]. *Hyalomma aegyptium* ticks occur in northern Africa, western Asia, and southern Europe and, apart from their preferential hosts, tortoises, the immature stages may alternatively feed on lizards, birds, and small- or medium-sized mammals [64,65]. Presumably, these vertebrates might influence expansion some pathogens associated with *H. aegyptium* beyond typical geographical areas. Since the ecology of *B. turcica* is not sufficiently known [66], the definite cause of detection of this bacterium in a *I. kaiseri* larva requires further studies.

Of the 14 *B. burgdorferi* s.l. species so far detected in Europe ([39,61,67],), 10 were detected in red-fox derived ticks, including three identified in tissues of these hosts. Among the 10 species found in ticks, six were discovered for the first time in North America, namely: *B. burgdorferi* s.s., *B. bissettiae*, *B. americana, B. carolinensis*, *B. californiensis,* and *B. lanei.* These species together with *B. kurtenbachii,* detected so far only in North America [68], and *B. finlandensis,* known solely from Europe [69], are the most closely related in the whole LD stock. To confirm and determine their proper identification we used three molecular markers, i.e., *flaB* and *p66* gene fragments and *mag*–*trnI* intergenic spacer. Regardless of the marker used, their examination confirmed less genetic distance value between the six species stated in North America and Europe than between the other four LD species detected in this study. A possible explanation why so many closely related species can exist on continents separated by the Atlantic Ocean may be the hypothesis of *Borrelia* evolution proposed by Estrada-Peña et al. [70]. The authors assume common evolution of the ancestors of all modern ticks and *Borrelia* species and species level-differentiation at the time of existence of the Pangea supercontinent (between 300 and 180 million years ago). Their contemporary occurrence is the effect of species expansion within Pangea and then separation of individual *Borrelia* species or populations transmitted by competent tick vectors as the result of the land masses moving apart and then forming the present continents. According to this hypothesis, it is not possible to delineate a “European” or “American” origin of the *B. burgdorferi* s.l. stock. Their common ancestor might evolve from the relapsing fever group before the Pangea supercontinent splitting and forming of Laurasia and Gondwana [70].

*Borrelia garinii* identified in red foxes, as well as in their ticks, showed a high level of diversity, which is characteristic of this species. However, unlike birds, carnivore mammals are not recognized as its reservoir. Studies of seabird colonies within the North Atlantic region revealed alongside local genetic variants of *B. garinii,* also the genotypes similar to those obtained from some human clinical samples in Europe [71]. The North Atlantic region is a particular habitat for *B. garinii* with the seabird tick *Ixodes uriae* [72]. Similarly, carnivore mammals, including foxes with their specific tick fauna species, might create conditions favorable to new genetic variant of this bacterium and the remaining LD *Borrelia* species detected in this study.

Except for the three most prevalent *B. burgdorferi* s.l. species (*B. garinii*, *B. afzelii*, and *B. burgdorferi* s.s.), the other seven species displayed a highly patchy distribution and were found in three, two or one of our sampling sites. Notably, the highest diversity of the rare spirochetes (i.e., *B. bissettiae, B. carolinensis, B. californiensis,* and *B. lanei*) was recorded in Niemierzewo Forest Range. It is located within Sierakowski Landscape Park with diverse ecosystems, including moraine hills, river valleys, dunes, lakes, arable fields, and various forest types. These heterogeneous ecosystems may account for the observed high diversity of *Borrelia* species, and the park may act as their biodiversity hotspot.

## 5. Conclusions

Our findings underscore the importance of burrow-dwelling *Ixodes* species in the ecology of *B*. *burgdorferi* s.l. in west-central Poland. The presence of 10 species of this complex in carnivore-associated *I. kaiseri* and *I. canisuga* indicates that these non-human biting ticks may serve as maintenance vectors in silent enzootic transmission cycles. Although their vectorial capacity remains unknown, they could potentially increase diversity of *Borrelia* species which may be transferred to the local population of the generalist tick *I. ricinus*. Detection in fox-derived samples, only three of the 10 spirochete species infecting engorged ticks, implies that these carnivores appear to be rather incompetent, or exhibit reduced and/or short-lived infectivity for most of them. It cannot be excluded that red foxes in the absence of widespread and long-term infections could serve as so-called “co-feeding transmission hosts” [73], especially that almost in half of the tick-infested animals the co-feeding events were observed. Nevertheless, competent reservoir species for these spirochete species remain unknown and we cannot come to any final conclusions. On the other hand, a distinct predominance of *B. garinii* in PCR-positive animals, as well as in infected fox-derived larval ticks, suggests that this species, along with *B. afzelii,* the second most prevalent species, may be preferentially associated with canids. It means that avian and mammalian-adapted spirochete species occupying different ecological niches might be concurrently maintained by red foxes.

## Figures and Tables

**Table 1:** Primers used for the amplification DNA of *Ixodes* ticks and *Borrelia* spirochetes.

Specificity	Genetic Marker	Sequence of Primers (5′->3′)	Anneling Temp. (°C)	Length of Amplicons (bp)	Usage	Reference
Ixodes *	*ITS2*	SP2-26F: CTTCCCGTGGCTTCGTCT	48	453–694	PCR-RFLP, sequencing	[21]
		SP2-1299R: CTATGCTTAAATTCAGGG				
		Nested PCR				
		SP2-100F: TCGTTTTGACTGTGTCGG	48			
		SP2-1274: CCTGATGTGAGGTCGACA				
	*coxI*	CO1-45F: ACTAACCATAAAGACACATTGG	44	706	sequencing	This study
		CO1-1100R: GAATTGGCTAAAATAATTCC				
		Nested PCR				
		CO1-375F: GGCAGGAACTGGATGAAC	47			
		CO1-1086R: AATTCCTGTTAATCCYCC				
Borrelia **	*flaB*	132f: TGGTATGGGAGTTTCTGG	56	604	PCR-RFLP, sequencing	[24]
		905r: TCTGTCATTGTAGCATCTTT				
		Nested PCR				
		220f: CAGACAACAGAGGGAAAT	54			
		823r: TCAAGTCTATTTTGGAAAGCACC				
		FL84F: AGAAGCTTTCTAGTGGGTACAGA	57	789	sequencing	This study
		FL976R: GATTGGCCTGTGCAATCAT				
		Nested PCR				
		FL120F: TGATGATGCTGCTGGGATGG	56			
		FL908R: TCATCTGTCATTGTAGCATCTT				
*Borrelia burgdorferi* sensu lato	*p66*	p66-226ldf—GGTACTACATATGCTTCTGT	54	596–599	sequencing	This study
		p66-1320htldr—AGGTACACTTCAATTTGGATACA				
		Nested PCR				
		p66-487ldf—CCTTTTTTGTTGTCTTCATAGC	53			
		p66-1087ldr—AATTCATCAATAACATACTCT				
Borrelia **	*mag—trnI*	glz199f—GTAAGTTTGCCAGGACCATT	56	309–1183	sequencing	This study
		ile20r—TGAACATCCGACCTCAGG				
		Nested PCR				
		glz435f—TAAGCTTCCGTTTCAAC	58			
		ile65r—CAGACCTGCGCTCTAACC				

* primers specific to the whole *Ixodes* genus. ** primers specific to the whole *Borrelia* genus, including Lyme disease borreliae (*B. burgdorferi* s.l.), RF borreliae, and REP borreliae.

**Table 2:** Occurrence of four *Ixodes* species collected from ears of 243 red foxes harvested in west-central Poland.

Tick Species	No. (%) Infested Hosts/No. Ticks/No. Ticks per Host/No. Ticks per Infested Host			
	Total	Females	Nymphs	Larvae
*I. ricinus*	56 (23)/162/0.7/2.9	28 (11.5)/43/0.2/1.5	31 (12.8)/58/0.2/1.9	23 (9.5)/61/0.3/2.7
*I. kaiseri*	82 (33.7)/1205/5/14.7	13 (5.3)/21/0.1/1.6	39 (16)/75/0.3/1.9	51 (21)/1109/4.6/21.7
*I. canisuga*	45 (18.5)/188/0.8/4.2	8 (3.3)/11/0.05/1.4	19 (7.8)/25/0.1/1.3	32 (13.2)/152/0.6/4.8
*I. hexagonus*	19 (7.8)/28/0.1/1.5	1 (0.4)/1/0.004/1.0	7 (2.9)/8/0.03/1.1	13 (5.3)/19/0.08/1.5
Total	120 (49.4)/1583/6.5/13.2	41 (16.9)/76/0.3/1.9	73 (30)/166/0.7/2.3	83 (34.2)/1341/5.5/16.2

**Table 3:** *Borrelia burgdorferi* s.l. (Bb s.l.) species identified by PCR-RFLP procedure in three different types of tissue samples (*n* = 558) obtained from 243 red foxes harvested in west-central Poland. Cumulated infection prevalence in the hosts is presented in the last line of the table.

Tissue Samples	No. Tested/	Bb s.l. Species ^a^ in Positive Tissue Samples (Animals) (% Prevalence)			
	Positive (%)	BG	BA	BSP	BG/BA
Blood	216/51 (23.6)	37 (72.5)	4 (7.8)		10 (19.6)
Skin (ear)	243/10 (4.1)	4 (40)	4 (40)	1 (10)	1 (10)
Liver	99/6 (6.1)	2 (33.3)	4 (66.7)		
Total *	558/67 (12)	43 (64.2)	12 (17.9)	1 (1.5)	11 (16.4) ^b^
Red foxes	(243/57 (23.5))	[36 (63.2)]	[4 (7)]	[1 (1.7)]	[16 (28.1)] ^c^

^a^ BG—*B. garinii*, BA—*B. afzelii*, and BSP—*B. spielmanii;* ^b^ BG/BA co-infection detected in a single tissue isolate type; ^c^ BG/BA co-infection found in one or two different tissue isolate types of the same host; * The number of infected tissue samples is higher than the number of infected foxes (67 vs. 57) because in 10 animals spirochetes were present concurrently in two tissue isolate types.

**Table 4:** Prevalence of *Borrelia* DNA in 943 feeding *Ixodes* ticks obtained from the ears of 243 red foxes harvested in west-central Poland.

Tick Species	No. (%) Hosts with at Least One Infected Tick	No. Tested/Infected (%)			
		Females	Nymphs	Larvae	Total
*I. ricinus*	26 (10.7)	43/19 (44.2)	58/21 (36.2)	61/17 (27.9)	162/57 (35.2)
*I. kaiseri*	50 (20.6)	21/9 (42.9)	75/33 (44)	479/131 (27.3)	575/173 (30.1)
*I. canisuga*	27 (11.1)	11/7 (63.6)	25/14 (56)	143/45 (31.5)	179/66 (36.9)
*I. hexagonus*	9 (3.7)	1/1 (100)	8/5 (62.5)	18/4 (22.2)	27/10 (37)
Total	81 (33.3)	76/36 (47.4)	166/73 (43.9)	701/197 (28.1)	943/306 (32.4)

**Table 5:** Prevalence of *Borrelia* species identified by PCR-RFLP procedure in 306 infected *Ixodes* ticks (Females/Nymphs/Larvae) collected from the ears of red foxes * in west-central Poland.

*Borrelia* spp.	No. (F/N/L) (%) of Ticks Infected				
	*I. ricinus*	*I. kaiseri*	*I. canisuga*	*I. hexagonus*	Total
*B. garinii*	13 (5/4/4) (22.8)	66 (4/13/49) (38.2)	17 (1/3/13) (25.8)	4 (1/2/1) (40.0)	100 (11/22/67) (32.7)
*B. afzelii*	20 (5/10/5) (35.1)	49 (0/12/37) (28.3)	20 (3/2/15) (30.3)	3 (0/2/1) (30.0)	92 (8/26/58) (30.1)
*B. burgdorferi s.s.*	2 (1/0/1) (3.5)	8 (0/1/7) (4.6)		2 (0/1/1) (20.0)	12 (1/2/9) (3.9)
*B. valaisiana*	1 (1/0/0) (1.8)	1 (0/0/1) (0.6)	1 (0/1/0) (1.5)		3 (1/1/1) (1.0)
*B. bissettiae*		4 (1/0/3) (2.3)	3 (1/2/0) (4.5)	1 (0/0/1) (10.0)	8 (2/2/4) (2.6)
*B. spielmanii*	1 (1/0/0) (1.8)	6 (0/0/6) (3.5)	2 (0/0/2) (3.0)		9 (1/0/8) (2.9)
*B. carolinensis*	8 (3/1/4) (14.0)	22 (2/3/17) (12.7)	6 (1/2/3) (9.1)		36 (6/6/24) (11.8)
*B. californiensis*	9 (1/6/2) (15.8)	6 (2/1/3) (3.5)	9 (0/2/7) (13.6)		24 (3/9/12) (7.8)
*B. lanei*		2 (0/0/2) (1.2)	3 (0/1/2) (4.5)		5 (0/1/4) (1.6)
*B. americana*	2 (2/0/0) (3.5)		1 (0/1/0) (1.5)		3 (2/1/0) (1.0)
*B. garinii/B. afzelii*	1 (0/0/1) (1.8)	5 (0/2/3) (2.9)	3 (0/0/3) (4.5)		9 (0/2/7) (2.9)
*B. garinii/B. burgdorferi s.s.*		1 (0/0/1) (0.6)	1 (1/0/0) (1.5)		2 (1/0/1) (0.7)
*B. garinii/B. americana*		1 (0/1/0) (0.6)			1 (0/1/0) (0.3)
*B. afzelii/B. burgdorferi s.s.*		1 (0/0/1) (0.6)			1 (0/0/1) (0.3)
*B. turcica* ** (REP)		1 (0/0/1) (0.6)			1 (0/0/1) (0.3)
Total	57 (19/21/17)	173 (9/33/131)	66 (7/14/45)	10 (1/5/4)	306 (36/73/197)

* A total of 18 (33.3%) of the 243 tested animals carried at least one infected tick. ** The member of the reptile-associated (REP) borreliae.

**Table 6:** Red foxes with at least one infected larva (F—female, N—nymph, L—larva).

Fox No.	Fox Infection	Borrelia Species in Ticks (F/N/L)									
		BG	BA	BB	BV	BSP	BBI	BCL	BCR	BAM	BLN
1	-	+/+/−	+/+/−	+/−/−		−/−/+ *	−/+/−				
2	BG ^a^/BA ^a^		−/−/+ **	−/−/+ *							
3	BG ^a^/BA ^a,b^	−/−/+ **	−/+/−								
4	BG ^a^/BA ^a,b^	+/−/+ ***								+/−/−	
5	-		−/−/+ *								
6	-		−/−/+ *								
7	-	−/+/+ ***	−/+/−								
8	-	−/−/+ *									
9	-	−/−/+ *	−/−/+ *								
10	-	−/+/+ ***									
11	-	−/−/+ *									
12	-	−/−/+ *	−/+/+ ***					−/−/+ *			−/−/+ *
13	-							+/+/−	−/−/+ *		
14	BA ^a,b^	−/−/+ *	−/−/+ *					−/+/+ ***	−/+/+ ***		
15	-								+/−/+ ***		
16	BG ^a^/BA ^a^	−/−/+ **	−/−/+ **					−/+/+ ***	+/−/+ ***		+/−/−
17	BG ^a^		−/−/+ *								
18	BG ^a^	−/+/−						−/−/+ *			
19	BG ^a^		−/−/+ *								
20	BG ^a^		−/−/+ *					−/−/+ *	−/−/+ *		
21	BG ^a^	−/−/+ **							−/+/−		
22	BG ^a^/BA ^a^							−/−/+ *	−/+/−		
23	BG ^a^		−/−/+ *			−/−/+ *			−/−/+ *		−/−/+ *
24	-	+/−/−	+/+/−	−/−/+ *							
25	-	−/−/+ *									
26	-	−/−/+ *									
27	-	−/−/+ *	−/+/+ ***								
28	-	−/−/+ *									
29	-	−/−/+ *					−/+/−				
30	-	−/−/+ *									
31	-	−/+/+ ***	−/−/+ *				−/−/+ *				
32	-	+/+/+ ***	+/+/+ ***	−/+/+ ***							
33	-	−/−/+ *		−/−/+ *							
34	-	−/−/+ *	−/−/+ *	−/−/+ *							
35	-	−/−/+ *									
36	-	−/+/+ ***	−/−/+ *	−/−/+ *	−/−/+ *	−/−/+ *	−/−/+ *		−/−/+ *		
37	-		+/−/+ ***					−/−/+ *	+/+/+ ***		
38	-					−/−/+ *	−/−/+ *	−/−/+ *	−/−/+ *		
39	-	−/−/+ *	−/−/+ *	−/−/+ *							
40	-	−/−/+ *	−/−/+ *	−/−/+ *		−/−/+ *			−/−/+ *		−/−/+ *
41	-	−/−/+ *	−/−/+ *								
42	-	−/−/+ *	−/−/+ *								
43	-	−/−/+ *	−/−/+ *	−/−/+ *							
44	-	−/−/+ *									
45	-	−/−/+ *									
46	-	−/−/+ *	−/−/+ *								

* Larva infected in the case of non-systemic fox infection. ** Possible larval infection by infected fox. *** Possible larval infection during co-feeding. ^a^—blood infection, ^b^—skin infection.

## Data Availability

All data generated or analysed during this study are included in this published article and its supplementary information file. The accession numbers of DNA sequences obtained for ticks and bacteria are mentioned in Material and Methods and are available in the GenBank (https://ww.ncbi.nlm.nih.gov/nuccore, accessed on 23 March 2018).

## Supplementary Materials

The following supporting information can be downloaded at: https://www.mdpi.com/article/10.3390/pathogens11060696/s1, Table S1. Seasonal occurrence of four Ixodes species collected from the ears of 243 red foxes harvested during the fox-hunting seasons (1 June to 31 March) between 2009 and 2011 in west-central Poland. Table S2. Detection of LD Borrelia species concurrently in two isolate types derived from red foxes. BA—*B. afzelii*, BG—*B. garinii*. Table S3. Comparison of the selected 112 partial flaB gene sequences of Borrelia species amplified from red foxes (blood, and skin samples: *n* = 13, and 2, respectively) and their engorged Ixodes ticks (*n* = 97) sampled in this study. Table S4. MEGA X results of mean distance between Borrelia species obtained on the basis of flaB gene sequence fragment comparison. Table S5. Comparison of the selected 33 partial p66 gene sequences of B. burgdorferi s.l. species amplified from engorged Ixodes ticks removed from red foxes sampled in this study. Table S6. MEGA X results of mean distance between B. burgdorferi s.l. species obtained on the basis of p66 gene sequence fragment comparison. Table S7. Comparison of the selected 44 partial intergenic spacer (IGS) of 3-methyladenine glycosylase (mag) and tRNA-Ile (trnI) genes sequences of Borrelia species amplified from engorged Ixodes ticks removed from red foxes sampled in this study. Table S8. MEGA X results of mean distance between Borrelia species obtained on the basis of intergenic spacer (IGS) of 3-methyladenine glycosylase (mag) and tRNA-Ile genes sequence fragment comparison.

## Institutional Review Broad Statement

The study was not conducted on live animals and none of the foxes was mortified for the purpose of the study. All foxes used for the study came from hunting conducted as planned wildlife management and were already dead while the tissues and blood were collected. Therefore, the Ethics Committee or Institutional Review Board approval is not required.
