# Supplementary material for: Red Foxes (Vulpes vulpes) Are Exposed to High Diversity of Borrelia burgdorferi Sensu Lato Species Infecting Fox-Derived Ixodes Ticks in West-Central Poland
Source: Pathogens. 2022 Jun 16;11(6):696. doi: 10.3390/pathogens11060696 (PMC9229790; doi:10.3390/pathogens11060696)
Supplement: Supplementary file 1 [file pathogens-11-00696-s001.zip › pathogens-1753305-supplementary.pdf]

Table S1. Seasonal occurrence of four *Ixodes* species collected from ears of 243 red foxes harvested during the fox-hunting seasons (June 1st to March 31st) between 2009 and 2011 in west-central Poland

Tick species	No. (%) infested hosts /no. ticks/no. ticks per host/n. ticks per infested host		
	Spring/Summer (VI-VIII) (*n* = 70)*	Autumn (IX-XI) (*n* = 96)	Winter (XII–III) (*n* = 77)
I. ricinus	29 (41.4)/102/1.5/3.5	17 (17.7)/43/0.4/2.5	10 (13.0)/17/0.2/1.7
I. kaiseri	21 (30.0)/383/5.5/18.2	28 (29.2)/290/3.0/10.4	33 (42.9)/532/6.9/16.1
I. canisuga	4 (5.7)/14/0.2/3.5	21 (21.9)/65/0.7/3.1	20 (30.0)/109/1.4/5.5
I. hexagonus	3 (4.3)/3/0.1/1.0	8 (8.3)/12/0.1/1.5	8 (10.4)/13/0.2/1.6
Total	33 (47.1) /502/7.2/15.2	41 (42.7) /410/4.3/10.0	46 (59.7) /671/8.7/14.6

*In square brackets: number of hosts sampled during a season.

Table S2. Detection of LD *Borrelia* species concurrently in two isolate types derived from red foxes. BA – *B. afzelii*, BG – *B. garinii*

Fox' no.	Isolate type		
	Blood	Skin	Liver
1	BA	BA/BG	-
2	BA/BG	BA	-
3	BG	BG	-
4	BG	BG	-
5	BA	BA	-
6	BG	BA	-
7	BG	-	BA
8	BG	-	BA
9	BG	-	BA
10	-	BG	BA

Table S3. Comparison of the selected 112 partial *flaB* gene sequences of *Borrelia* species amplified from red foxes (blood, and skin samples: *n* = 13, and 2, respectively) and their engorged *Ixodes* ticks (*n* = 97) sampled in this study.

Identified *Borrelia* species	Strain	Accession number	Reference sequence (species, strain)	Accession number	Query coverage	E value	Identity
B. garinii	9N1L-IC	KF422759	*B. garinii* 20047	CP028861	100.0%	0.0	789/789 (100.0%)
	1N1N-IH	KF422762			100.0%	0.0	789/789 (100.0%)
	4C1F-IH	KF422765			100.0%	0.0	788/789 (99.87%)
	K2-1F-IR	KF422774			100.0%	0.0	788/789 (99.87%)

	1N1L-IC	KF422779			100.0%	0.0	787/789 (99.75%)
	3N5L-IC	KF422780			100.0%	0.0	787/789 (99.75%)
	7N5L-IC	KF422764			100.0%	0.0	786/789 (99.62%)
	15Z13L-IC	KF422781			100.0%	0.0	786/789 (99.62%)
	33NM1934-LC	MG944996			100.0%	0.0	786/789 (99.62%)
	1N2N-IC	KF422763			100.0%	0.0	781/789 (98.99%)
	3A2F-IC	KF422758			100.0%	0.0	777/789 (98.48%)
	8N1L-IC	KF422782			100.0%	0.0	777/789 (98.48%)
	1A1F-IR	KF422766			100.0%	0.0	775/789 (98.23%)
	2Z2F-IR	KF422767			100.0%	0.0	772/789 (97.85%)
	3A2N-IH	HM802184			100.0%	0.0	602/604 (99.67%)
	AM10g-BL*	KF422841			100.0%	0.0	601/604 (99.50%)
	28K2N2-IC	KF918603			100.0%	0.0	601/604 (99.50%)
	9PD12L1-IR	KF918612			100.0%	0.0	600/604 (99.34%)
	34M11g-BL*	KF422840			100.0%	0.0	598/604 (99.01%)
	30C10g-BL*	KF422844			100.0%	0.0	598/604 (99.01%)
	22W56L1-IC	KF918609			100.0%	0.0	598/604 (99.01%)
	12M10g-BL*	KF422838			100.0%	0.0	597/604 (98.84%)
	39PA2L10g-IK	KF422846			100.0%	0.0	596/604 (98.68%)
	32M11-BL*	KF422839			100.0%	0.0	595/604 (98.51%)
	8N2L-IC	KF918607			100.0%	0.0	595/604 (98.51%)
	4M4L-IC	HM802182			100.0%	0.0	594/604 (98.34%)
	7N4L-IC	KF422817			100.0%	0.0	594/604 (98.34%)
	10C10g1-BL*	KF422842			100.0%	0.0	594/604 (98.34%)
	5N17L1-IC	KF918606			100.0%	0.0	594/604 (98.34%)
	8N9L2-IC	KF918608			100.0%	0.0	594/604 (98.34%)
	29C4N1-IC	KF918611			100.0%	0.0	594/604 (98.34%)
	3N13L2-IC	KF918604			100.0%	0.0	593/604 (98.18%)
	5P1N-IR	HM802188			100.0%	0.0	592/604 (98.01%)
	10C10g2-BL*	KF422843			100.0%	0.0	592/604 (98.01%)
	28K2N1-IC	KF918602			100.0%	0.0	592/604 (98.01%)
	22W56L2-IC	KF918610			100.0%	0.0	592/604 (98.01%)
	7Z8L-IH	HM802185			100.0%	0.0	591/604 (97.85%)
	2Z-SK**	KF918600			100.0%	0.0	591/604 (97.85%)
	6A1N-IC	KF918601			100.0%	0.0	590/604 (97.68%)
	15Z2L-IC	KF918605			100.0%	0.0	589/604 (97.52%)
	36K3L10-IC	KF422845			100.0%	0.0	585/604 (96.85%)
B. afzelii	15Z1L-IC	KF422794	*B. afzelii* BO23	CP018262	100.0%	0.0	789/789 (100.0%)
	22NM1779-LC	MG944961			100.0%	0.0	788/789 (99.87%)
	34MR2062-FR	MG944963			100.0%	0.0	788/789 (99.87%)
	15Z8L-IC	KF422795			100.0%	0.0	787/789 (99.75%)
	20MR2041-LR	MG944962			100.0%	0.0	787/789 (99.75%)
	7N1N-IC	KF422789			100.0%	0.0	786/789 (99.62%)
	28K1N-IH	KF422790			100.0%	0.0	786/789 (99.62%)
	3A1F-IK	KF422787			100.0%	0.0	784/789 (99.37%)

Identified *Borrelia* species	Strain	Accession number	Reference sequence (species, strain)	Accession number	Query coverage	E value	Identity
	8N10L-IC	KF422796			100.0%	0.0	784/789 (99.37%)
	1A10N-IR	KF422791			100.0%	0.0	781/789 (98.99%)
	3N13L1-IC	KF918614			100.0%	0.0	601/604 (99.50%)
	9PD12L2-IR	KF918616			100.0%	0.0	601/604 (99.50%)
	3A3F-IC	HM802193			100.0%	0.0	600/604 (99.34%)
	30C10a-BL*	KF422863			100.0%	0.0	600/604 (99.34%)
	42DG2N10-IK	KF422865			100.0%	0.0	600/604 (99.34%)
	8N9L1-IC	KF918615			100.0%	0.0	600/604 (99.34%)
	AM10a-BL*	KF422861			100.0%	0.0	599/604 (99.17%)
	39PA2L10a-IK	KF422864			100.0%	0.0	596/604 (98.68%)
	9M10-BL*	KF422858			100.0%	0.0	594/604 (98.34%)
	28C10-BL*	KF422862			100.0%	0.0	593/604 (98.18%)
	34M11a-BL*	KF422860			100.0%	0.0	591/604 (97.85%)
	12M10a-BL*	KF422859			100.0%	0.0	589/604 (97.52%)
B. burgdorferi s.s.	22NM1783-LC	MG944978	*B. burgdorferi* ZS7	CP001205	100.0%	0.0	787/789 (99.75%)
	22NM1798-LC	MG944979			100.0%	0.0	787/789 (99.75%)
	24NM1809-FR	MG944980			100.0%	0.0	787/789 (99.75%)
	31NM1824-NH	MG944981			100.0%	0.0	787/789 (99.75%)
	32NM1835-LH	MG944982			100.0%	0.0	787/789 (99.75%)
	32NM1843-LC	MG944983			100.0%	0.0	787/789 (99.75%)
	5N11N-IC	KF422802			100.0%	0.0	786/789 (99.62%)
	1A3L-IR	KF422799			100.0%	0.0	778/789 (98.61%)
	3A2F-IC	HM802191			100.0%	0.0	601/604 (99.50%)
	5N17L2-IC	KF918617			100.0%	0.0	601/604 (99.50%)
B. valaisiana	22NM1795-LK	MT118979	*B. valaisiana* VS116	NZ_ABCY02000001	100.0%	0.0	789/789 (100.0%)
	38MR2081-NK	MT118980			100.0%	0.0	789/789 (100.0%)
B. spielmanii	6MR2001-FR	MG944976	*B. spielmanii* A14S	ABKB02000003	100.0%	0.0	787/789 (99.75%)
	43MR2086-LK	MG944977			100.0%	0.0	787/789 (99.75%)
	22NM1790-LK	MT118981			100.0%	0.0	786/789 (99.62%)
	30NM1817-LK	MT118982			100.0%	0.0	786/789 (99.62%)
	L32-EA**	JF732881			100.0%	0.0	604/604 (100.0%)
B. bissettiae	22NM1781-LC	MG944964	*B. bissetiae* DN127	CP002746	100.0%	0.0	788/789 (99.87%)
	3A5N-IK	KF422807			100.0%	0.0	787/789 (99.75%)

	12N1N-IK	KF422806			100.0%	0.0	785/789 (99.49%)
	3N2L-IC	KF918618			100.0%	0.0	603/604 (99.83%)
B. carolinensis	22NM1786-LC	MG944970	*B. carolinensis* SCW-22	KF422810	100.0%	0.0	789/789 (100.0%)
	30NM1816-LC	MG944971			100.0%	0.0	789/789 (100.0%)
	30NM1818-LK	MG944972			100.0%	0.0	789/789 (100.0%)
	33NM1932-LC	MG944973			100.0%	0.0	789/789 (100.0%)
	33MR2061-LC	MG944974			100.0%	0.0	789/789 (100.0%)
	35MR2063-NK	MG944975			100.0%	0.0	789/789 (100.0%)
B. californiensis	23NM1807-LC	MG944984	*B. californiensis* CA446	KF422809	100.0%	0.0	789/789 (100.0%)
	5MR1999-LR	MG944986			100.0%	0.0	788/789 (99.87%)
	6MR2005-NR	MG944987			100.0%	0.0	788/789 (99.87%)
	13MR2025-LR	MG944988			100.0%	0.0	788/789 (99.87%)
	20MR2034-NK	MG944989			100.0%	0.0	788/789 (99.87%)
	20MR2038-LK	MG944991			100.0%	0.0	788/789 (99.87%)
	30MR2046-LK	MG944992			100.0%	0.0	788/789 (99.87%)
	37MR2080-LK	MG944995			100.0%	0.0	788/789 (99.87%)
	20MR2035-NK	MG944990			100.0%	0.0	788/789 (99.87%)
	33MR2048-LK	MG944993			100.0%	0.0	788/789 (99.87%)
	30NM1820-LC	MG944985			100.0%	0.0	785/789 (99.49%)
	33MR2051-LK	MG944994			100.0%	0.0	784/789 (99.37%)
B. americana	29C4N2-IC	KF918619	*B. americana* CA8	KF422811	100.0%	0.0	604/604 (100.0%)
	30C4F1-IR	KF918620			100.0%	0.0	603/604 (99.83%)
	30C4F2-IR	KF918622			100.0%	0.0	602/604 (99.67%)
	32C7N-IK	KF918621			100.0%	0.0	600/604 (99.34%)
B. lanei	33NM1921-LC	MG944965	*B. lanei* CA28	KF422812	100.0%	0.0	789/789 (100.0%)
	33NM1953-LC	MG944966			100.0%	0.0	789/789 (100.0%)
	5MR1995-LK	MG944967			100.0%	0.0	789/789 (100.0%)
	20NM2032-NK	MG944968			100.0%	0.0	789/789 (100.0%)
	43MR2088-LK	MG944969			100.0%	0.0	789/789 (100.0%)

B. turcica	33NM1854-LC	MG944997	*B. turcica* IST7	CP028884	100.0%	0.0	787/789 (99.75%)

* blood sample, ** skin sample

Table S4. MEGA X results of mean distance between *Borrelia* species obtained on the basis of *flaB* gene sequence fragment comparison.

	BB	BLN	BAM	BCL	BCR	BBI	BA	BSP	BG	BV	BTC
B. burgdorferi s.s.											
B. lanei	0.023										
B. americana	0.035	0.025									
B. californiensis	0.037	0.023	0.039								
B. carolinensis	0.037	0.020	0.043	0.030							
B. bissettiae	0.040	0.024	0.046	0.033	0.006						
B. afzelii	0.066	0.056	0.069	0.067	0.058	0.065					
B. spielmanii	0.064	0.051	0.071	0.066	0.055	0.062	0.057				
B. garinii	0.075	0.054	0.067	0.069	0.061	0.062	0.066	0.064			
B. valaisiana	0.059	0.041	0.057	0.052	0.045	0.051	0.053	0.062	0.062		
B. turcica	0.230	0.213	0.225	0.227	0.219	0.226	0.236	0.240	0.245	0.210	

BB: *B. burgdorferi* s.s., BLN: *B. lanei*, BAM: *B. americana*, BCL: *B. californiensis*, BCR: *B. carolinensis*, BCR: *B. carolinensis*, BBI: *B. bissettiae* , BA: *B. afzelii*, BSP: *B. spielmanii*, BG: *B. garinii*, BV: *B. valaisiana*, BTC: *B. turcica*

Table S5. Comparison of the selected 33 partial *p66* gene sequences of *B. burgdorferi* s.l. species amplified from engorged *Ixodes* ticks removed from red foxes sampled in this study.

Identified *Borrelia* species	Strain	Accession number	Reference sequence (species, strain)	Accession number	Query coverage	E value	Identity
B. garinii	4C1F-IH	MT118983	*B. garinii* 20047	CP028861	100.0%	0.0	599/599 (100.0%)
	33NM1934-LC	MT118984			100.0%	0.0	586/599 (97.83%)
B. afzelii	22NM1779-LC	MT118985	*B. afzelii* BO23	CP018262	100.0%	0.0	589/599 (98.33%)
	34MR2062-FR	MT118987			100.0%	0.0	592/599 (98.83%)
	20MR2041-LR	MT118986			100.0%	0.0	592/599 (98.83%)
B. burgdorferi	22NM1783-LC	MT118988	*B. burgdorferi* ZS7	CP001205	100.0%	0.0	589/596 (98.83%)
	22NM1798-LC	MT118989			100.0%	0.0	594/596 (99.66%)
	24NM1809-FR	MT118990			100.0%	0.0	589/596 (98.83%)

	32NM1843-LC	MT118991			100.0%	0.0	595/596 (99.83%)
B. valaisiana	22NM1795-LK	MT118992	*B. valaisiana* VS116	NZ_ABCY02000001	100.0%	0.0	594/599 (99.17%)
	38MR2081-NK	MT118993			100.0%	0.0	599/599 (100.0%)
B. spielmanii	6MR2001-FR	MT118996	*B. spielmanii* A14S	ABKB02000003	100.0%	0.0	598/599 (99.83%)
	43MR2086-LK	MT118997			100.0%	0.0	596/599 (99.50%)
	22NM1790-LK	MT118994			100.0%	0.0	597/599 (99.67%)
	30NM1817-LK	MT118995			100.0%	0.0	597/599 (99.67%)
B. bissettiae	22NM1781-LC	MT118998	*B. bissetiae* DN127	CP002746	100.0%	0.0	590/599 (98.50%)
B. carolinensis	30NM1816-LC	MT118999	*B. carolinensis* SCW-22	MT119003	100.0%	0.0	597/599 (99.67%)
	30NM1818-LK	MT119000			100.0%	0.0	589/599 (98.33%)
	33NM1932-LC	MT119001			100.0%	0.0	598/599 (99.83%)
	33MR2061-LC	MT119002			100.0%	0.0	591/599 (98.66%)
B. californiensis	23NM1807-LC	MT119004	*B. californiensis* CA446	MT119008	100.0%	0.0	595/599 (99.33%)
	30NM1820-LC	MT119005			100.0%	0.0	595/599 (99.33%)
	6MR2005-NR	MT119006			100.0%	0.0	593/599 (99.00%)
	33MR2051-LK	MT119007			100.0%	0.0	593/599 (99.00%)
B. americana	29C4N2-IC	MT119015	*B. americana* CA8	MT119019	100.0%	0.0	593/599 (99.00%)
	30C4F1-IR	MT119016			100.0%	0.0	592/599 (98.83%)
	30C4F2-IR	MT119017			100.0%	0.0	595/599 (99.33%)
	32C7N-IK	MT119018			100.0%	0.0	598/599 (99.83%)
B. lanei	33NM1921-LC	MT119009	*B. lanei* CA28	MT119014	100.0%	0.0	596/599 (99.50%)
	33NM1953-LC	MT119010			100.0%	0.0	596/599 (99.50%)
	5MR1995-LK	MT119011			100.0%	0.0	596/599 (99.50%)
	20NM2032-NK	MT119012			100.0%	0.0	595/599 (99.33%)
	43MR2088-LK	MT119013			100.0%	0.0	597/599 (99.67%)

Table S6. MEGA X results of mean distance between *B. burgdorferi* s.l. species obtained on the basis of *p66* gene sequence fragment comparison.

	BB	BLN	BAM	BCL	BCR	BBI	BA	BSP	BG	BV
B. burgdorferi s.s.										
B. lanei	0.046									
B. americana	0.051	0.053								
B. californiensis	0.069	0.068	0.096							
B. carolinensis	0.079	0.075	0.092	0.074						
B. bissettiae	0.073	0.068	0.086	0.070	0.033					
B. afzelii	0.082	0.086	0.089	0.103	0.104	0.086				
B. spielmanii	0.082	0.084	0.088	0.105	0.099	0.083	0.012			
B. garinii	0.088	0.096	0.100	0.119	0.108	0.097	0.054	0.056		
B. valaisiana	0.088	0.096	0.104	0.112	0.121	0.101	0.070	0.075	0.070	

BB: *B. burgdorferi* s.s., BLN: *B. lanei*, BAM: *B. americana*, BCL: *B. californiensis*, BCR: *B. carolinensis*, BCR: *B. carolinensis*, BBI: *B. bissettiae* , BA: *B. afzelii*, BSP: *B. spielmanii*, BG: *B. garinii*, BV: *B. valaisiana*, BTC: *B. turcica*

Table S7. Comparison of the selected 44 partial intergenic spacer (IGS) of 3-methyladenine glycosylase (*mag*) and tRNA-Ile (*trnI*) genes sequences of *Borrelia* species amplified from engorged *Ixodes* ticks removed from red foxes sampled in this study.

Identified *Borrelia* species	Strain	Accession number	Reference organism (species, strain)	Accession number	Query coverage	E value	Identity
B. garinii	4C1F-IH	MT119020	*B. garinii* 20047	CP028861	100.0%	0.0	1179/1183 (99.66%)
	15Z13L-IC	MT119021			100.0%	0.0	1176/1183 (99.41%)
	33NM1934-LC	MT119023			100.0%	0.0	1162/1183 (98.22%)
	8N1L-IC	MT119022			100.0%	0.0	1173/1183 (99.15%)
B. afzelii	22NM1779-LC	MT119027	*B. afzelii* BO23	CP018262	100.0%	0.0	784/793 (98.87%)
	34MR2062-FR	MT119029			100.0%	0.0	784/793 (98.87%)
	20MR2041-LR	MT119028			100.0%	0.0	784/793 (98.87%)
	7N1N-IC	MT119025			100.0%	0.0	783/793 (98.74%)
	28K1N-IH	MT119026			100.0%	0.0	783/793 (98.74%)
	3A1F-IK	MT119024			100.0%	0.0	783/793 (98.74%)
B. burgdorferi	22NM1783-LC	MT119030	*B. burgdorferi* ZS7	CP001205	100.0%	0.0	530/531 (99.81%)
	22NM1798-LC	MT119031			100.0%	0.0	529/531 (99.62%)
	24NM1809-FR	MT119032			100.0%	0.0	527/531 (99.25%)

	31NM1824-NH	MT119033			100.0%	0.0	530/531 (99.81%)
	32NM1843-LC	MT119034			100.0%	0.0	529/531 (99.62%)
B. valaisiana	22NM1795-LK	MT119035	*B. valaisiana* VS116	NZ_ABCY02000001	100.0%	0.0	861/861 (100.0%)
	38MR2081-NK	MT119036			100.0%	0.0	861/861 (100.0%)
B. spielmanii	6MR2001-FR	MT119039	*B. spielmanii* A14S	ABKB02000003	100.0%	0.0	497/498 (99.80%)
	43MR2086-LK	MT119040			100.0%	0.0	498/498 (100.0%)
	22NM1790-LK	MT119037			100.0%	0.0	496/498 (99.60%)
	30NM1817-LK	MT119038			100.0%	0.0	498/498 (100.0%)
B. bissettiae	22NM1781-LC	MT119042	*B. bissetiae* DN127	CP002746	100.0%	0.0	509/514 (99.03%)
	3A5N-IK	MT119041			100.0%	0.0	514/514 (100.0%)
B. carolinensis	22NM1786-LC	MT119043	*B. carolinensis* SCW-22	MT119049	100.0%	0.0	450/456 (98.68%)
	30NM1816-LC	MT119044			100.0%	0.0	451/456 (98.90%)
	30NM1818-LK	MT119045			100.0%	0.0	456/456 (100.0%)
	33NM1932-LC	MT119046			100.0%	0.0	455/456 (99.78%)
	33MR2061-LC	MT119047			100.0%	0.0	452/456 (99.12%)
	35MR2063-NK	MT119048			100.0%	0.0	450/456 (98.68%)
B. californiensis	23NM1807-LC	MT119050	*B. californiensis* CA446	MT119055	100.0%	0.0	402/403 (99.75%)
	30NM1820-LC	MT119051			100.0%	0.0	402/403 (99.75%)
	6MR2005-NR	MT119052			100.0%	0.0	403/403 (100.0%)
	33MR2051-LK	MT119053			100.0%	0.0	402/403 (99.75%)
	37MR2080-LK	MT119054			100.0%	0.0	403/403 (100.0%)
B. americana	29C4N2-IC	MT119062	*B. americana* CA8	MT119066	100.0%	0.0	534/536 (99.63%)
	30C4F1-IR	MT119063			100.0%	0.0	536/536 (100.0%)
	30C4F2-IR	MT119064			100.0%	0.0	530/536 (98.88%)
	32C7N-IK	MT119065			100.0%	0.0	532/536 (99.25%)

B. lanei	33NM1921-LC	MT119056	*B. lanei* CA28	MT119061	100.0%	0.0	485/488 (99.39%)
	33NM1953-LC	MT119057			100.0%	0.0	485/488 (99.39%)
	5MR1995-LK	MT119058			100.0%	0.0	486/488 (99.59%)
	20NM2032-NK	MT119059			100.0%	0.0	487/488 (99.80%)
	43MR2088-LK	MT119060			100.0%	0.0	487/488 (99.80%)
B. turcica	33NM1854-LC	MT119067	*B. turcica* IST7	CP028884	100.0%	0.0	308/309 (99.68%)

Table S8. MEGA X results of mean distance between *Borrelia* species obtained on the basis of intergenic spacer (IGS) of 3-methyladenine glycosylase (*mag*) and *tRNA-Ile* genes sequence fragment comparison.

	BB	BLN	BAM	BCL	BCR	BBI	BA	BSP	BG	BV	BTC
B. burgdorferi s.s.											
B. lanei	0.080										
B. americana	0.087	0.074									
B. californiensis	0.144	0.103	0.136								
B. carolinensis	0.130	0.133	0.153	0.128							
B. bissettiae	0.155	0.131	0.163	0.129	0.091						
B. afzelii	0.258	0.240	0.273	0.190	0.276	0.299					
B. spielmanii	0.278	0.235	0.280	0.186	0.270	0.310	0.113				
B. garinii	0.225	0.203	0.202	0.146	0.224	0.255	0.178	0.144			
B. valaisiana	0.222	0.183	0.230	0.170	0.251	0.270	0.275	0.186	0.290		
B. turcica	0.539	0.570	0.543	0.575	0.597	0.542	0.581	0.631	0.544	0.578	

BB: *B. burgdorferi* s.s., BLN: *B. lanei*, BAM: *B. americana*, BCL: *B. californiensis*, BCR: *B. carolinensis*, BCR: *B. carolinensis*, BBI: *B. bissettiae* , BA: *B. afzelii*, BSP: *B. spielmanii*, BG: *B. garinii*, BV: *B. valaisiana*, BTC: *B. turcica*
